# Pericardial fat pad plombage for pulmonary cavity causing massive air leakage

**DOI:** 10.1186/s40792-020-00917-7

**Published:** 2020-07-01

**Authors:** Tomoyuki Nakano, Hiroyoshi Tsubochi, Kentaro Minegishi, Shunsuke Endo

**Affiliations:** 1 Department of General Thoracic Surgery, Saitama Medical Center, Jichi Medical University, Saitama, Japan; 2 Department of General Thoracic Surgery, Jichi Medical University, 3311-1 Yakushiji, Shimotsuke, Tochigi, 329-0498 Japan

**Keywords:** Pleural fistula, Pericardial fat pad, Refractory pneumothorax

## Abstract

**Background:**

Secondary pneumothorax after chemotherapy for a malignant pulmonary tumor is a complication from a large cavity causing refractory pneumothorax.

**Case presentation:**

A 61-year-old man was referred due to prolonged air leakage from a pulmonary cavity that developed after treatment for pulmonary metastases from renal cell carcinoma. As air leakage continued after thoracic drainage and endobronchial occlusion, we planned thoracoscopy-assisted surgery. Intraoperatively, a large cavity opening to the pulmonary cavity was found in the left upper lobe. As it was difficult to repair the fistula using staplers or direct sutures because the pleura around the cavity was thick and hard, we attempted to plombage the cavity with a pericardial fat pad. After the operation, air leakage immediately disappeared and no recurrence of the pneumothorax was found.

**Conclusion:**

This novel method can be useful to seal a large bronchopleural fistula that causes refractory pneumothorax.

## Background

Secondary pneumothorax after chemotherapy for a malignant pulmonary tumor is a well-known complication that sometimes occurs [1]. Molecular-targeted agents can cause malignant tumors to markedly regress, and as a result, a large cavity opening to the pleural cavity can develop that causes refractory pneumothorax. We herein report a new method to treat pneumothorax due to a large cavity with a bronchopleural fistula, which occurred after treatment of pulmonary metastases from renal cell carcinoma with molecular-targeted agents and immune checkpoint inhibitors (ICIs).

## Case presentation

**Fig. 1:**
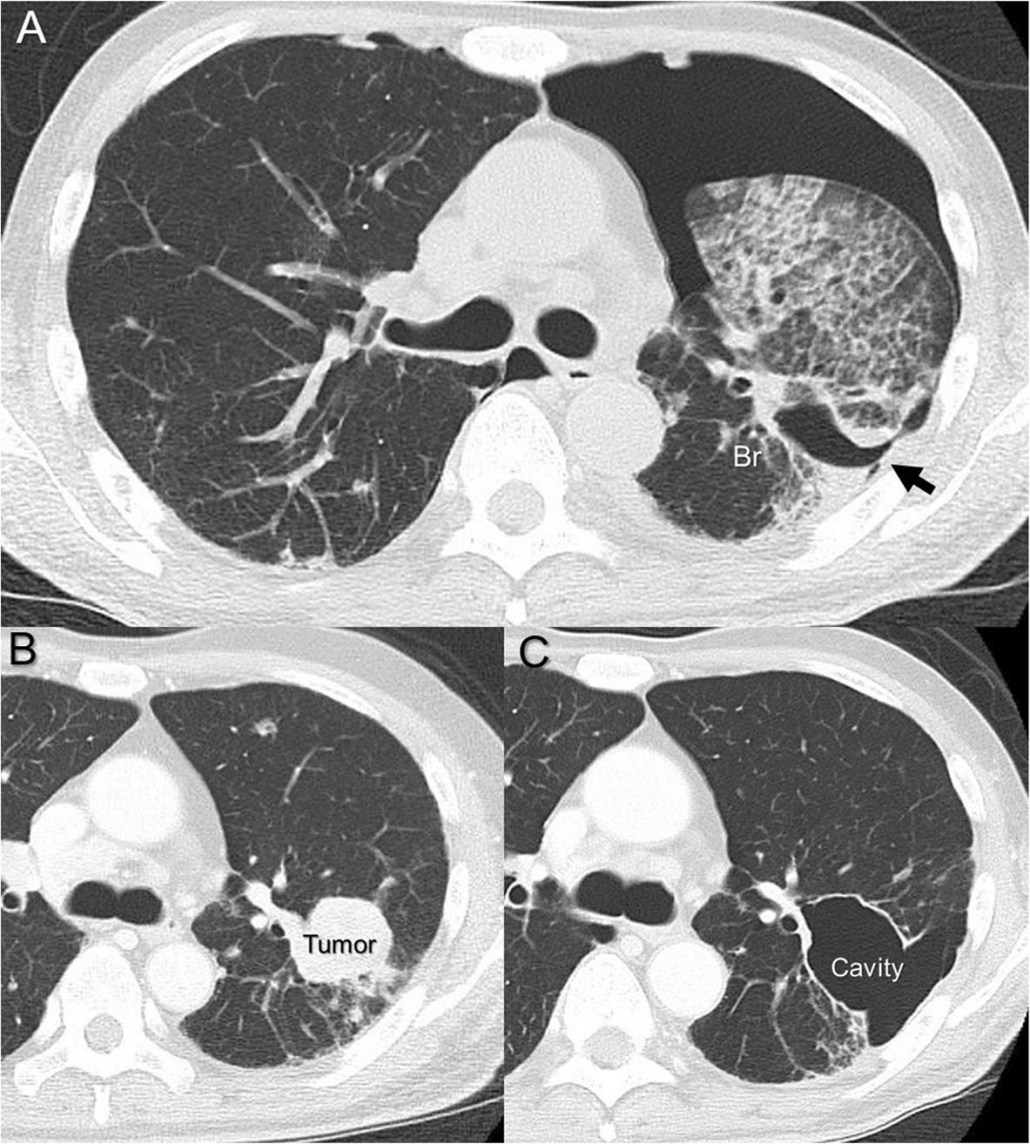
Chest computed tomography (CT) revealing left pneumothorax and a pleural defect (black arrow) in the left upper lobe (LUL) (**a**). CT before axitinib therapy initiation showing a nodule in the LUL (**b**). CT after the therapy showing a cavity that developed at the same site as the tumor (**c**). Br, bronchus

A 61-year-old man was referred to our hospital because of dyspnea. He had multiple pulmonary metastases from renal cell carcinoma (RCC) and had previously been treated with molecular-targeted agents and ICIs (pazopanib, sunitinib, nivolumab, and axitinib gradually). Chest X-ray revealed bilateral pneumothorax and chest tube drainage was performed. Chest computed tomography (CT) demonstrated a pulmonary cavity in the left upper lobe (LUL) (Fig. 1a). A CT prior to treatment revealed a nodule 3 cm in diameter, and a follow-up CT demonstrated cavity development at the same site that had developed during treatment with Axitinib, a vascular endothelial growth factor receptor-tyrosine kinase inhibitor (VEGFR-TKI) (Fig. 1b, c). Major air leakage continued for 1 week after chest tube drainage had been performed. Even after occlusion of the segmental bronchus (left B^1 + 2^) with an Endobronchial Watanabe Spigot was performed using a flexible bronchoscope, air leakage continued for 10 days. At this point, we performed thoracoscopy-assisted surgery under general anesthesia. Since in our case the bronchopleural fistula was large on CT and direct suturing may have been difficult, we planned to fill the cavity with a pericardial fat pad (PFP), which is often used as a sealant to control air leaks during pulmonary resection.

**Fig. 2:**
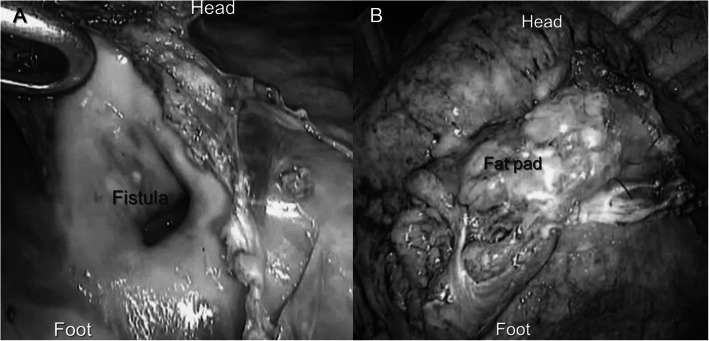
Intraoperative findings (top, cranial side; left, ventral side). A huge fistula with a cavity was found in the LUL via left-side thoracoscopy (**a**). Via mini-thoracotomy assisted by thoracoscopy, the cavity was filled by a harvested pedicle pericardial fat pad (PFP), which was secured with sutures and fibrin glue (**b**)

During the operation, a huge cavity with a pleural defect was found in the LUL (Fig. 2a, Video 1). It was difficult to repair the pleural defect using staples or direct sutures as the pleura around the cavity was thick and hard. Therefore, we harvested a pedicle pericardial fat pad (PFP) using an ultrasonic scalpel and used it to fill the cavity, which was sutured with the thick pleura around the cavity using a 4-0 absorbable monofilament suture (Fig. 2b). Prior to this maneuver, the cavity was sprayed with fibrin glue. Finally, we sutured the thickened fibrous tissue adhered to the parietal pleura near the cavity in order to assure sealing and PFP fixation. Next, the pulmonary air leak was unclear via right-side thoracoscopy, and we extensively scratched the parietal pleura to provoke adhesion.

**Fig. 3:**
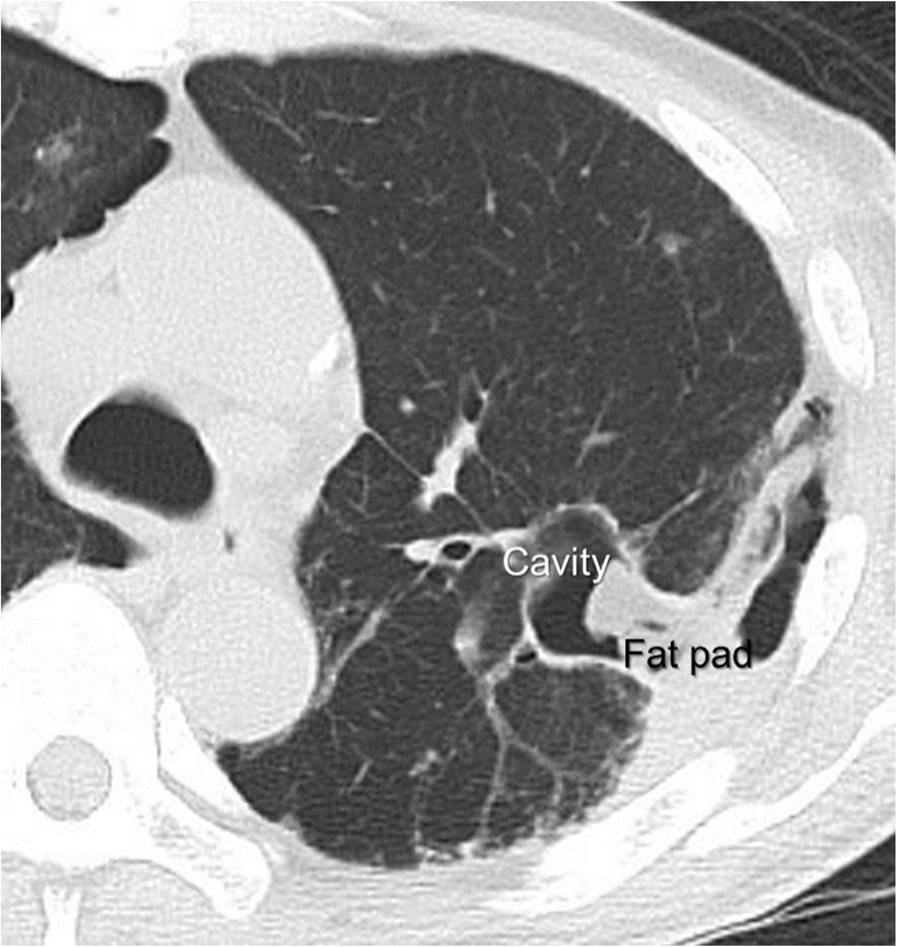
Postoperative chest CT demonstrated that the pulmonary cavity was occluded by the PFP

After the operation, the air leakage from the left lung immediately disappeared and recurrence was not seen. The right pneumothorax was recovered after pleurodesis with OK-432. Chest CT at 3 months after the surgery demonstrated that the pulmonary cavity was occluded by the PFP (Fig. 3).

**Additional file 1: Video 1** Intraoperative findings (top, cranial side; left, ventral side). A large cavity with a pleural defect was found in the LUL and the visceral pleura around the cavity became thicker. A pedicle pericardial fat pad was harvested using an ultrasonic scalpel and then inserted and fixed with sutures and fibrin glue to the visceral pleura around the cavity. Finally, the thickened pleura around the cavity was sutured in order to assure sealing and PFP fixation.

## Discussion

Primary mechanisms have been proposed to explain spontaneous secondary pneumothorax in patients with pulmonary metastasis [2]. Therapy-related tumor necrosis of subpleural nodules with bronchopleural fistula formation is the most likely mechanism. Secondary therapy-related refractory pneumothorax may increase because of the dramatic response by molecular targeted therapies. In one study, the development of pneumothorax associated with the use of axitinib was reported to have occurred in 4 (1.1%) of 359 RCC patients that had received axitinib [3].

The volume of PFP we obtained was sufficient to fill the cavity, and we attempted not only to cover the orifice but also to fill the cavity completely. Therefore, we named this method “fat pad plombage.” An air space developed in the bottom of the cavity after surgery probably because of ventilation; therefore, the PFP should be fixed on the bottom through suturing. Although the efficacy of free PFP for sealing intraoperative alveolar air leaks has been reported [4, 5], usefulness of PFP to seal a large pulmonary bronchopleural fistula causing refractory pneumothorax, as observed in our case, has not been reported. In this case, we used pedicled PFP; however, free PFP is also thought to be useful. Indeed, the use of PFP in previous reports as a sealant for preventing alveolar air leaks was not pedicled but free [4, 5]. If the cavity and fistula were apart from the anterior mediastinum and pedicled PFP was not reachable, free PFP would be chosen. In our case, bronchial occlusion with EWS before surgery could not stop air leakage completely; however, it might play a role in the success of treatment by reduction of massive leakage. EWS occlusion prior to the surgery may be a strategy to treat similar cases.

Reportedly, PFP produces neovascularization in chick chorioallantoic membranes and cytokines related to tissue repair, such as interleukin-1α and interleukin-1β, tumor necrosis factor-α, and interleukin-6 [6]. Pedicled PFP may be more beneficial than free PFP since angiogenesis of the cavity wall might be inhibited by axitinib in our case. No infection around the fistula was observed in our case. We have enough data to assess whether or not this method can be used in infectious cases. Although early atrophy of the fat pad may occur, the usefulness of free subcutaneous fat pads as a sealant for alveolar air leakage was confirmed on CT performed 6 months after surgery [7]. In addition, histologic examination showed that the fat structure was maintained 1 month after surgery, although the feeding vessel to the fat mass was not observed in a canine model [4]. In our case, the follow-up period was insufficient, and a longer follow-up is preferable to assess our method. Further accumulation of cases is necessary to evaluate the validity of this method.

## Conclusion

This case study suggested that plombage of the pleural cavity connecting to the pleural space using a PFP is effective to recover from refractory pneumothorax.

## Data Availability

There is no available data and materials to be shared.

## Abbreviations

ICI: Immune checkpoint inhibitor
RCC: Renal cell carcinoma
CT: Computed tomography
LUL: Left upper lobe
VEGFR-TKI: Vascular endothelial growth factor receptor-tyrosine kinase inhibitor
PFP: Pericardial fat pad
Br: Bronchus
